# Small GTP-binding protein Rho-mediated signaling promotes proliferation of rheumatoid synovial fibroblasts

**DOI:** 10.1186/ar1694

**Published:** 2005-02-18

**Authors:** Shingo Nakayamada, Hitoshi Kurose, Kazuyoshi Saito, Akira Mogami, Yoshiya Tanaka

**Affiliations:** 1 First Department of Internal Medicine, University of Occupational and Environmental Health, School of Medicine, Fukuoka, Japan; 2 Department of Pharmacology and Toxicology, Graduate School of Pharmaceutical Sciences, Kyushu University, Fukuoka, Japan; 3 Pharmaceuticals Research Unit, Research & Development Division, Mitsubishi Pharma Corporation, Yokohama, Japan

## Abstract

Rho is a major small GTP-binding protein that is involved in the regulation of various cell functions, including proliferation and cell migration, through activation of multiple signaling molecules in various types of cells. We studied its roles in synovial fibroblasts (SFs) in patients with rheumatoid arthritis (RA) and clarified its relevance to RA synovitis, with the following results. 1)We found that the thrombin receptor was overexpressed on RA synovial fibroblasts (RA SFs) and that thrombin induced a marked proliferation and progression of the cell cycle to the S phase in these cells. 2)We also found that thrombin efficiently activated Rho. 3)Rho activation and proliferation and the progression of the cell cycle to the S phase were completely blocked by p115RGS (an N-terminal regulator of the G-protein signaling domain of p115RhoGEF) and by the C-terminal fragments of Gα13 (an inhibitor of the interaction of receptors with G13). 4)Thrombin induced the secretion of IL-6 by RA SFs, but this action was blocked by p115RGS or Gα13. Our findings show that the actions of thrombin on the proliferation of RA SFs, cell-cycle progression to the S phase, and IL-6 secretion were mainly mediated by the G13 and RhoGEF pathways. These results suggest that p115RGS and Gα13 could be potent inhibitors of such functions. A rational design of future therapeutic strategies for RA synovitis could perhaps include the exploitation of the Rho pathway to directly reduce the growth of synovial cells.

## Introduction

Rheumatoid arthritis (RA) is characterized by synovial proliferation, neovascularization, and accumulation of inflammatory cells in inflamed joints. Synovial cells are markedly activated by cytokines, adhesion molecules, and coagulation factors, resulting in hyperplasia of the synovial tissue, and the activated synovial cells produce inflammatory cytokines and degradative enzymes. These pathological processes in RA synoviocytes are tightly regulated by intracellular signaling. The small GTPase Rho is a pivotal regulator of several signaling pathways, including the remodeling of actin cytoskeleton, transcriptional regulation, and cell-cycle progression [1-4]. Like other regulatory GTPases, Rho requires GDP/GTP exchange dependent on guanine nucleotide exchange factors (GEFs) for its activation [5]. GEFs are critical regulators of Rho activation and thereby control a variety of cellular responses such as cell proliferation and cytokine production. However, the relevance of Rho-mediated signaling to inflammatory processes in RA is largely unknown.

Among the various stimuli that activate the GEF–Rho pathway, thrombin is the best-known activator through the following sequence of events: binding thrombin to protease-activated receptor-1 including a thrombin receptor; activation of heterotrimeric G proteins Gq, Gi, and G12/13 [6-8]; activation of p115RhoGEF by the α subunit of G12/13; binding of a Rho-specific GEF containing a Dbl homology domain to Rho; and activation of Rho by GDP/GTP exchange [9-12].

Recent studies have indicated that Rho regulates cellular functions in inflammatory cells [13-16]. Rho GTPases have been implicated in the regulation of cell proliferation and IL-2 production in T cells [13,17,18]. RA is a representative inflammatory disease and is characterized by accumulation of T cells and proliferation of synovial fibroblasts [19,20]. Although many molecules, including inflammatory cytokines such as IL-1, tumor necrosis factor (TNF), and IL-6 and growth factors, have been implicated as pathogenic factors in RA, the coagulation system is also involved in the inflammatory processes in RA synovitis. High levels of various clotting and fibrinolytic factors such as thrombin are found in the synovial fluid of patients with RA [21-24], and high concentrations of thrombin are detected in RA synovial tissue [25,26]. Moreover, thrombin promotes chemotaxis and adhesion of inflammatory cells such as lymphocytes and the production of various proinflammatory molecules [25,27,28]. Thrombin may therefore play an important pathological role in RA synovitis.

The aim of the present study was to determine the role of Rho-mediated signaling in the regulation of synovial proliferation and cytokine production in RA SFs. The results indicate that thrombin stimulation induces proliferation and IL-6 secretion by RA SFs through G13 and Rho pathways and suggest that the G13–GEF–Rho pathway plays an important role in the RA inflammatory process.

## Materials and methods

The study protocol was approved by the Human Ethics Review Committee of the University of Occupational and Environmental Health, Japan, and we obtained a signed consent form from each subject before taking tissue samples used in the present study.

### Synovial tissues and culture of synovial fibroblasts

Synovial tissues were obtained from five women (aged 45 to 66 years) with active RA or osteoarthritis (OA) whose disease had been diagnosed according to the criteria of the American College of Rheumatology [29-32] and who were treated by joint replacement surgery. All the enrolled patients with RA had more than six swollen joints, more than three tender joints, and an erythrocyte sedimentation rate (Westergren) of >28 mm/hour.

Samples were dissected under sterile conditions in PBS and were immediately prepared for culture of fibroblast-like synovial cells. Briefly, the tissue samples were minced into small pieces and digested with collagenase (Sigma Aldrich, Tokyo, Japan) in serum-free DMEM (Gibco BRL, Grand Island, NY, USA). The cells were filtered through a nylon mesh and then were washed extensively and suspended in DMEM supplemented with 10% FCS (Bio-Pro, Karlsruhe, Germany) and streptomycin/penicillin (10 units/ml; Sigma Aldrich). Finally, isolated cells were seeded in 25-cm^2 ^culture flasks (Falcon, Lincoln Park, NJ, USA) and cultured in a humidified 5% CO_2 _atmosphere. After overnight culture, nonadherent cells were removed and incubation of adherent cells was continued in fresh medium. At confluence, the cells were trypsinized, passaged at a 1:3 split ratio, and recultured. The medium was changed twice each week, and the cells were used after 2 to 5 passages. We characterized cultured synovial cells derived from the synovium of RA patients. The cells were spindle-shaped and grew in a cobblestone pattern. Flow cytometric analysis of these cells indicated that they lacked macrophage markers such as major-histocompatibility-complex class II antigens CD14 and CD11b (data not shown). Thus, RA synovial cells are type B synovial-fibroblast-like cells.

### Materials

Human thrombin was purchased from Sigma Aldrich. The following mAbs were used: fluorescein-isothiocyanate-conjugated control mAb anti-Thy 1.2 (Becton Dickinson, San Jose, CA, USA) and antithrombin receptor mAb ATAP2 (Santa Cruz Biotechnology, Santa Cruz, CA, USA). A Rho activation kit containing glutathione S-transferase (GST)-Rhotekin-Rho-binding domain (RBD) (GST-RBD) beads was purchased from Cytoskeleton (Denver, CO, USA).

### Adenoviral infection

Recombinant adenoviruses encoding green-fluorescent protein (GFP), the C-terminal regions of Gα12 (Gα12-ct), the C-terminal regions of Gα13 (Gα13-ct), and the regulator of the G-protein signaling domain of p115RhoGEF (p115RGS) were produced as described previously [33]. RA SFs were plated onto a six-well culture dish and cultured in DMEM containing 10% FCS. After 24 hours, the cells were infected with recombinant adenoviruses at a multiplicity of infection of 30 for 1 hour at 37°C. Cells infected or not infected with adenovirus were then starved in DMEM with 1% FCS and cultured for an additional 48 hours before treatment. Under these conditions, infection with adenoviruses coding for GFP made almost 100% of cells GFP-positive. None of these vectors produced cytotoxic effects on RA SFs until 96 hours after infection, as confirmed by trypan blue staining (data not shown).

### Flow microfluorometry

Staining and flow-cytometric analysis of RA SFs were conducted by standard procedures, as described previously [34], using a FACScan (Becton Dickinson, Mountain View, CA, USA). Briefly, cells (2 × 10^5^) were incubated with fluorescein-isothiocyanate-conjugated negative control mAb anti-Thy-1.2 or antithrombin receptor mAb at saturating concentrations in fluorescence-activated cell sorter (FACS) medium consisting of Hanks' balanced salt solution (Nissui, Tokyo, Japan), 0.5% human serum albumin (Mitsubishi Pharma, Osaka, Japan), and 0.2% NaN_3 _(Sigma Aldrich) for 30 min at 4°C. After three washes in FACS medium, the cells were analyzed with the FACScan. Cell-surface antigens on single cells were quantified using standard beads, QIFKIT (Dako Japan, Kyoto, Japan), as described previously [35,36]. The data were used to construct the calibration curve of mean fluorescence intensity versus antibody-binding capacity. The cell specimen was analyzed on the FACScan and antibody-binding capacity calculated by interpolation on the calibration curve. When green-fluorescence laser detection was set at 450 nm in the FACScan used, antibody-binding capacity = 414.45 × exp (0.0092 × Mean fluorescence intensity) (*R*2 = 0.9999). Subsequently, specific antibody-binding capacity was obtained after corrections for background, apparent antibody-binding capacity of the negative control mAb anti-Thy-1.2. The specific antibody-binding capacity is the mean number of accessible antigenic sites per cell, referred to as antigen density and expressed in sites/cell.

### Proliferation assay

RA SFs (1 × 10^4^) infected with or free of adenoviruses were seeded and incubated in 96-well flat-bottomed microfilter plates (Costar, Cambridge, MA, USA) in DMEM containing 1% FCS for 48 hours at 37°C and were then stimulated with the indicated amount of thrombin. At 24 hours after the thrombin stimulation, cells were stained with TetraColor One (Seikagaku, Tokyo, Japan) including tetrazolium and an electron-carrier mixture for detecting cell proliferation. After the cells had been stained in this way for 1 hour at 37°C, the optical density value of each well was measured using an ELISA plate reader at 450 nm.

### Cell-cycle analysis

RA SFs infected with or free of adenoviruses were cultured for 48 hours in DMEM containing 1% FCS and then stimulated with the indicated amount of thrombin. At 24 hours after thrombin stimulation, the cells were collected, washed with PBS, and fixed in 70% ethanol for 2 hours at 4°C. After treatment of cells with 10 μg/ml ribonuclease (Wako, Osaka, Japan) for 15 min at 37°C, the cells were stained with 50 μg/ml propidium iodide (Sigma Aldrich) for 2 minutes. The DNA content was subsequently measured by FACScan.

### Rho activation assay

Rho activation was determined by a pull-down assay using GST-RBD beads [37,38]. Forty-eight hours after adenovirus infection, RA SFs were stimulated with 10 units/ml thrombin, quickly washed with ice-cold Tris-buffered saline, and lysed in 500 μl of lysis buffer (50 mM Tris, pH 7.5, 10 mM MgCl_2_, 0.5 M NaCl, 1% Triton X-100, 0.5% sodium deoxycholate, 0.1% SDS, 500 μg/ml tosyl arginine methyl ester, and 10 μg/ml each of leupeptin and aprotinin). Cell lysates were immediately centrifuged at 8,000 rpm at 4°C for 5 min and equal volumes of lysates were incubated with 30 μg GST-RBD beads for 1 hour at 4°C. The beads were washed twice with wash buffer (in mmoles: 25 Tris, pH 7.5; 30 MgCl_2_; 40 NaCl), and bound Rho was eluted by boiling each sample in Laemmli sample buffer. Eluted samples from the beads and total cell lysate were then electrophoresed on 12% SDS–PAGE gels, transferred to nitrocellulose, blocked with 5% nonfat milk, and analyzed by western blotting using a polyclonal anti-Rho antibody.

### Statistical analysis

Data are expressed as mean ± standard deviation (SD) of the number of indicated patients. Differences from the control were examined for statistical significance by the Mann–Whitney *U *test. A *P *value less than 0.05 denoted the presence of a statistically significant difference.

## Results

### High expression of thrombin receptor on RA SFs

**Figure 1:**
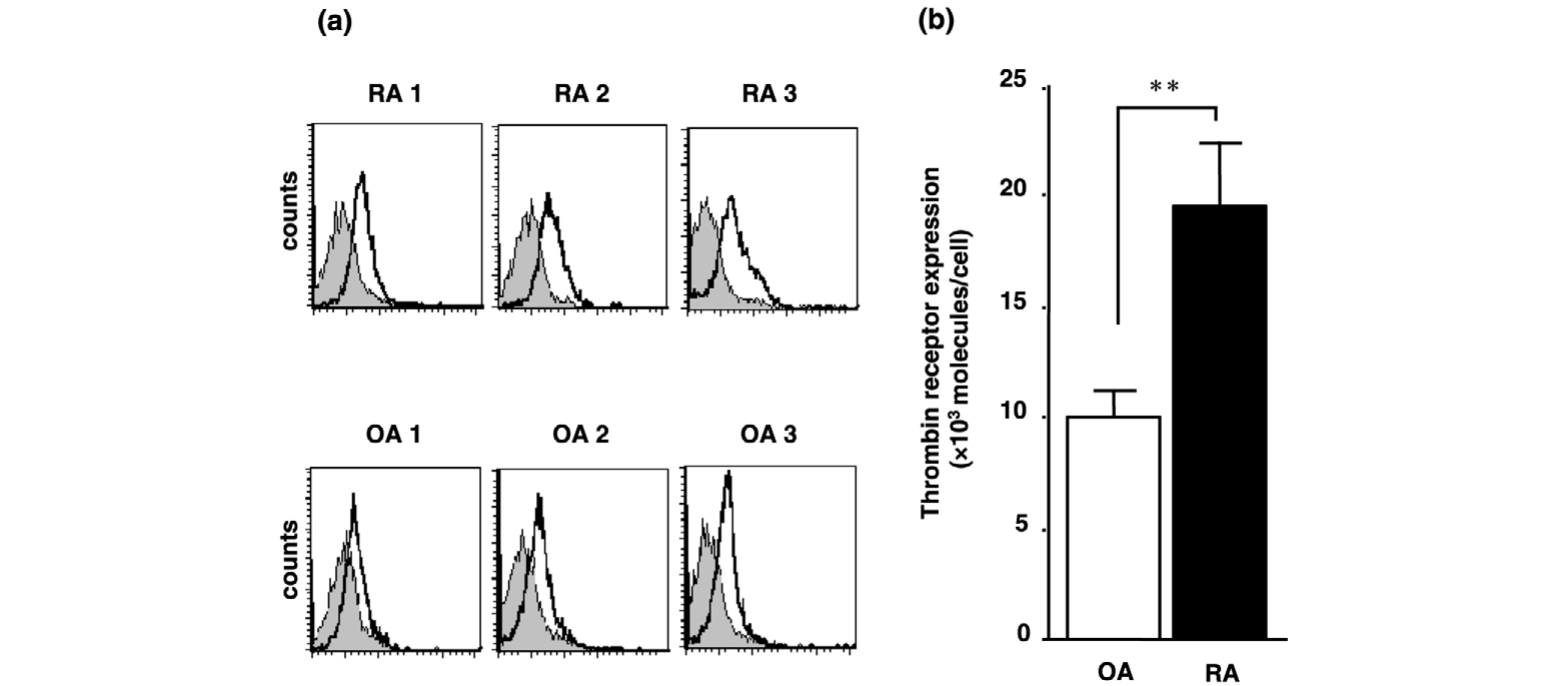
Overexpression of thrombin receptor on SFs from patients with RA. **(a) **Histograms representing thrombin receptor expression on synovial fibroblasts (SFs) from rheumatoid arthritis (RA) and osteoarthritis (OA) patients. Cells were stained with the antithrombin receptor mAb ATAP2. Flow-cytometric analyses were performed using FACScan. Open histograms represent the number of cells stained with ATAP2 in each logarithmic scale on a fluorescence amplifier. Shaded histograms represent profiles of anti-Thy1.2 mAb as a negative control. **(b) **Comparison of thrombin receptor expression in RA and OA SFs. The expression of thrombin receptor was analyzed by FACScan. Each value represents the number of molecules expressed per cell, calculated using standard QIFKIT beads from five similar experiments, as described in Materials and methods. Data are expressed as mean ± standard deviation for five independent donors. FACS, fluorescence-activated cell sorter. **P < 0.01.

First, we assessed the expression of thrombin receptor on synovial fibroblasts using FACScan. Fig. 1a shows the histogram of thrombin receptor expressed on RA and OA synovial fibroblasts. Although thrombin receptor was expressed on both types of synovial fibroblasts, its level was significantly higher in RA than OA fibroblasts (Fig. 1b). These results were identified in synovial fibroblasts from patients with RA and OA (*n *= 5 each).

### Thrombin induces synovial proliferation and S-phase progression of the cell cycle in RA SFs

**Figure 2:**
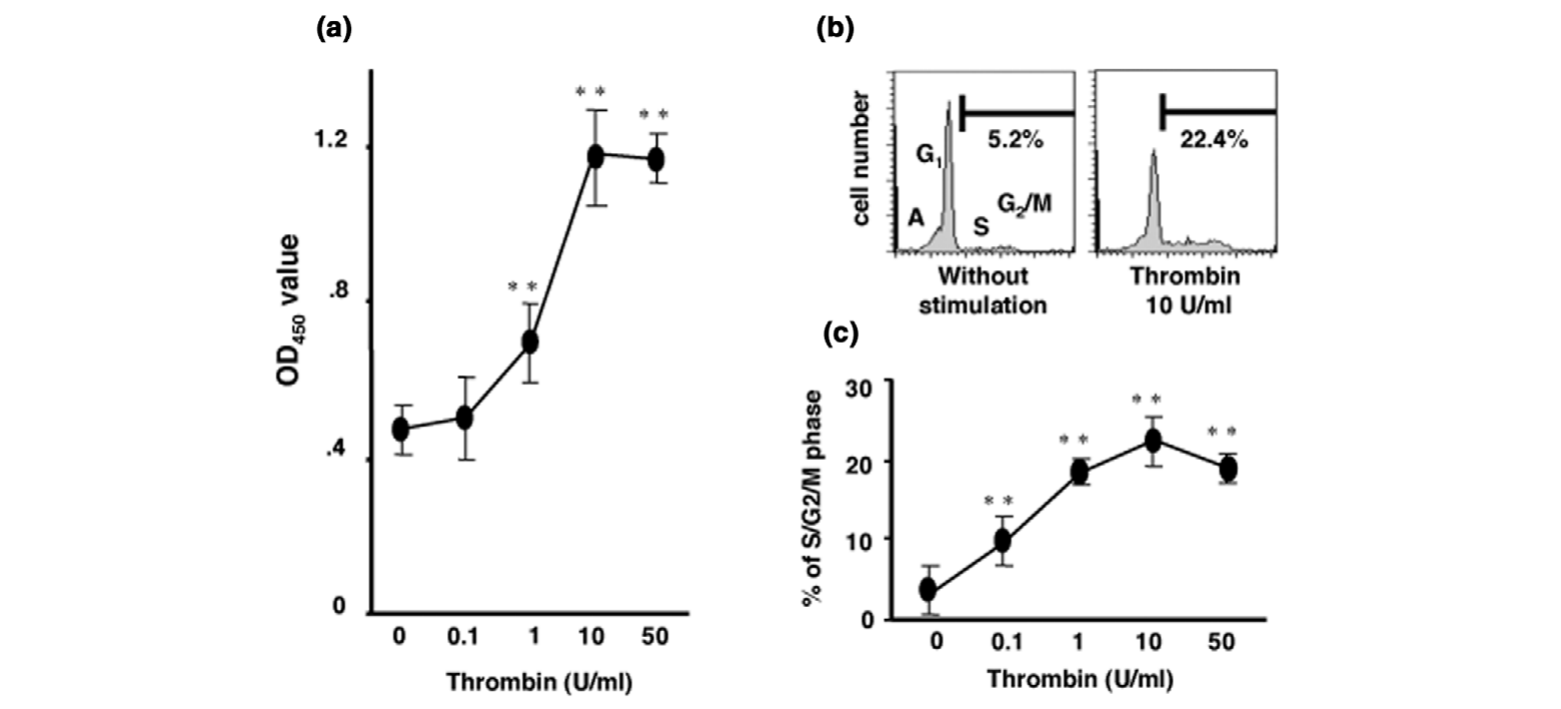
Thrombin induces synovial proliferation and progression to S phase in RA SFs. Cells were cultured for 48 hours in DMEM containing 1% FCS and then stimulated with the indicated amount of thrombin. For the proliferation assay, at 24 hours after thrombin stimulation, cells were stained with TetraColor One including tetrazolium and electron-carrier mixture for detecting cell proliferation. The optical density (OD) was measured by ELISA plate reader at 450 nm. For analysis of the cell cycle, at 24 hours after thrombin stimulation, cells were collected, washed with PBS, and fixed in 70% ethanol for 2 hours at 4°C. After treatment of cells with 10 μg/ml ribonuclease for 15 min at 37°C, fixed cells were stained with 50 μg/ml propidium iodide for 2 min. The DNA content was subsequently measured by FACScan fluorescence-activated cell sorter. **(a) **Dose-dependent proliferation of synovial fibroblasts (SFs) from rheumatoid arthritis (RA) patients. The OD was measured by ELISA plate reader at 450 nm. **(b) **Histogram representing the cell cycle in RA SFs, as detected by FACScan. **(c) **Dose-dependent S-phase progression of the cell cycle in RA SFs. Numbers represent the percentage of cells exhibiting mean channel fluorescence (FL2-H) in the S/G_2_/M phase of the cell. Data are expressed as mean ± standard deviation for five experiments, using five independent donors. **P < 0.01 in comparison with the value found without thrombin stimulation.

To assess the effect of thrombin on the proliferation of RA SFs, we performed proliferation assay and cell-cycle analysis. After cells were starved for 48 hours in DMEM containing 1% FCS, cells were stimulated with the indicated amount of thrombin for 24 hours. Thrombin significantly induced cell proliferation in a dose-dependent manner (Fig. 2a). As shown in Fig. 2b, the vast majority of the starved cells existed at the propidium-iodide-low G0/G1 phase and showed little progression to S phase. However, thrombin significantly increased the S/G_2_/M phase of the cell in a dose-dependent manner (Fig. 2b,c). The maximum effects of thrombin on cell proliferation and progression to S phase were noted at 10 units/ml. These results indicate that thrombin acts as an important stimulator of RA synovial proliferation.

**Figure 3:**
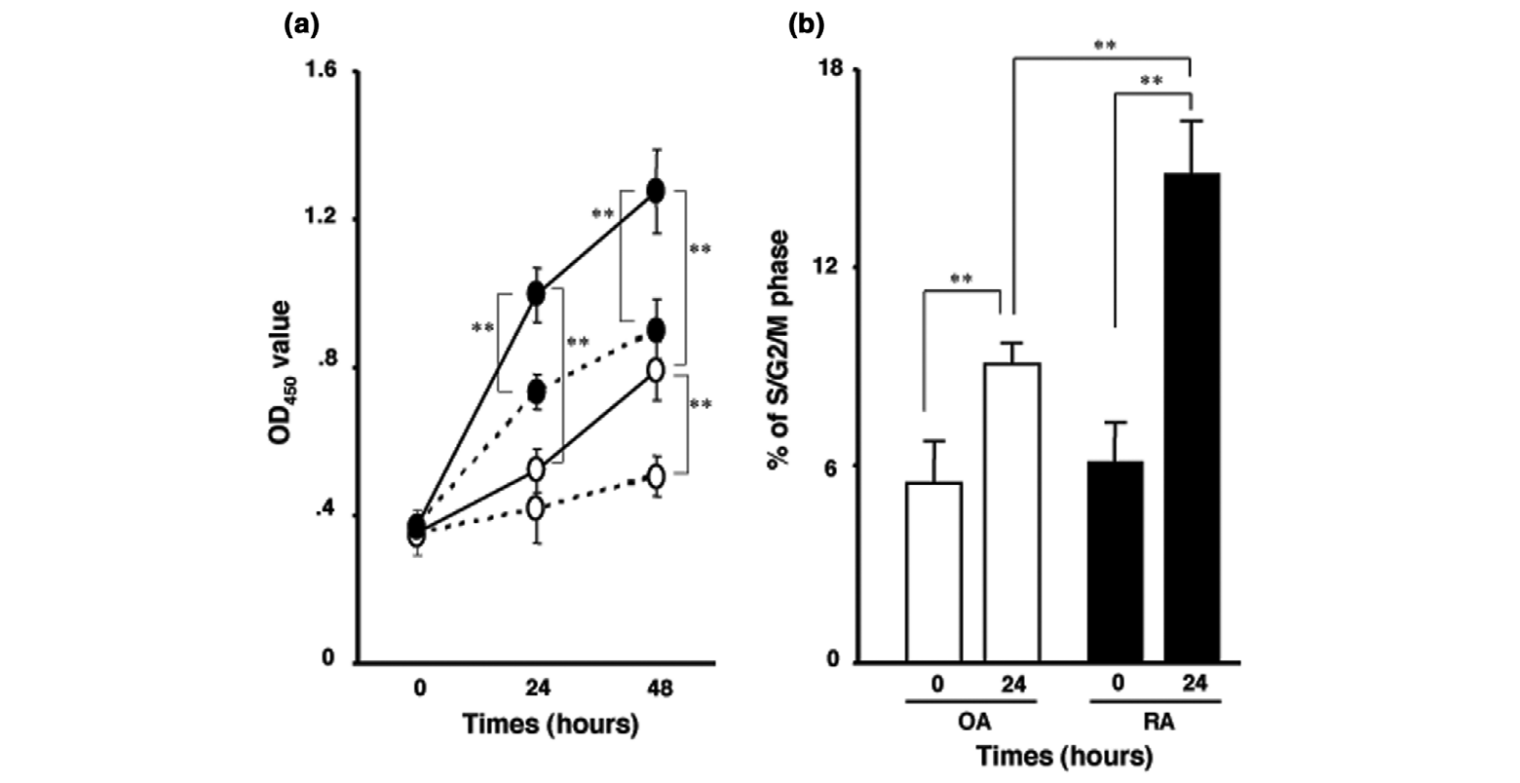
Effects of thrombin on proliferation and cell cycle in SFs from RA and OA. Cells were cultured for 48 hours in DMEM containing 1% FCS and then stimulated with 10 units/ml thrombin. **(a) **Time-course of proliferation of synovial fibroblasts (SFs) from patients with rheumatoid arthritis (RA) (dark symbols) and patients with osteoarthritis (OA) (light symbols). Continuous lines, thrombin-stimulated cells; dotted lines, unstimulated cells. The optical density (OD) was measured by ELISA plate reader at 450 nm. **(b) **Comparison of cell cycle in RA SFs and OA SFs. Numbers represent the percentage of cells exhibiting mean channel fluorescence (FL2-H) in the S/G_2_/M phase of the cell-division cycle. Data are expressed as mean ± standard deviation of five experiments, using five independent donors. **P < 0.01.

We next compared cell growth and the cell cycle of RA SFs with those of OA SFs. As shown in Fig. 3a, thrombin induced cell growth and cell-cycle progress in both RA and OA SFs, whereas unstimulated cells did not proliferate well. However, thrombin-induced proliferation of RA SFs was significantly higher than that of OA SFs after incubation for 24 or 48 hours. As shown in Fig. 3b, thrombin induced cell-cycle progress to S phase in both RA and OA SFs, but the responses of RA SFs were significantly higher than those of OA SFs after 24 hours of incubation.

### Involvement of small GTP-binding protein Rho activation in thrombin-induced signaling in RA SFs

**Figure 4:**
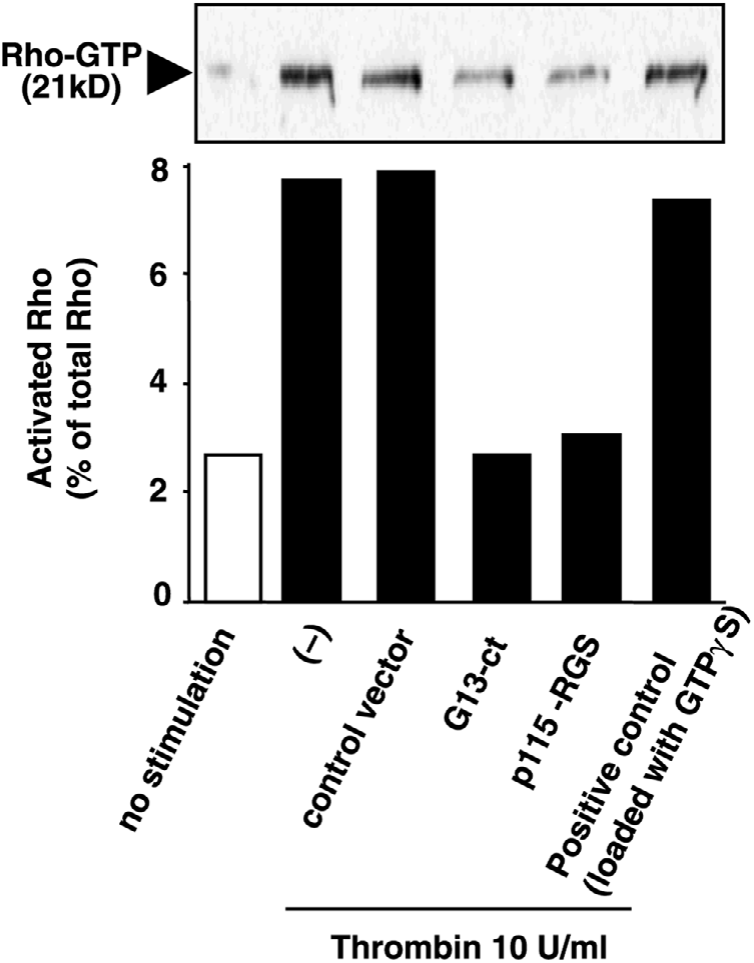
Inhibition of thrombin-induced Rho activation by expression of Gα13-ct and p115RGS in RA synovial fibroblasts. Rheumatoid arthritis (RA) synovial fibroblasts (SFs) were or were not infected with adenoviruses encoding green-fluorescent protein (GFP) (control vector), the C-terminal regions of Gα13 (Gα13-ct), or P115RGS. Cells that were not infected with adenovirus were incubated in the medium alone. Cells were then cultured for 48 hours in DMEM containing 1% FCS, then stimulated with 10 units/ml thrombin for 1 minute or were loaded with GTPγS (positive control), after which they were lysed to measure Rho activity. Rho activity is indicated by the amount of Rho bound by the Rhotekin-Rho-binding domain (RBD) **(top)**. The percentage of activated Rho **(graph) **is expressed as a ratio relative to 4% of total Rho (4% of total protein used in the RBD bead pull-down experiments). Results are representative of three experiments. Western blot analysis confirmed that equal amounts of total Rho were used for the pull-down assay under each condition (data not shown). (-), cells without infection; p115RGS, regulator of G-protein signaling domain of p115Rho guanine nucleotide exchange factor.

Thrombin is known to bind to protease-activated receptor-1 such as thrombin receptor, and binding of thrombin to receptors leads to activation of the G protein Gα13 and induces activation of p115RhoGEF, a Rho-specific GEF, and thereby activates Rho [9-12]. We measured Rho activation and its inhibition in RA SFs using the GST-Rhotekin fusion protein. We used the C-terminal regions of Gα12 and Gα13, which inhibit Gα12 and Gα13 from coupling with each receptor, or the regulator of the G-protein signaling domain of p115RhoGEF, which inhibits endogenous p115RhoGEF function by blocking the interaction of p115RhoGEF with Gα12/13 and by its GTPase-activating-protein activity on Gα12/13 [33,39,40]. After adenoviral infection, cells were stimulated with 10 units/ml thrombin, and the lysates from cells were incubated with Rhotekin bound to GST beads to determine Rho activation. As shown in Fig. 4, thrombin stimulation increased the amount of activated Rho in RA SFs infected or not infected with adenoviruses encoding control vector, suggesting that thrombin activates Rho in RA SFs. However, the expression of Gα13-ct or p115RGS completely prevented thrombin-induced Rho activation (Fig. 4). These results suggest that Rho is involved in signaling via thrombin-stimulation, which leads to synovial proliferation.

### Involvement of G protein Gα13 and Rho-GEF in thrombin-induced proliferation of RA SFs

**Figure 5:**
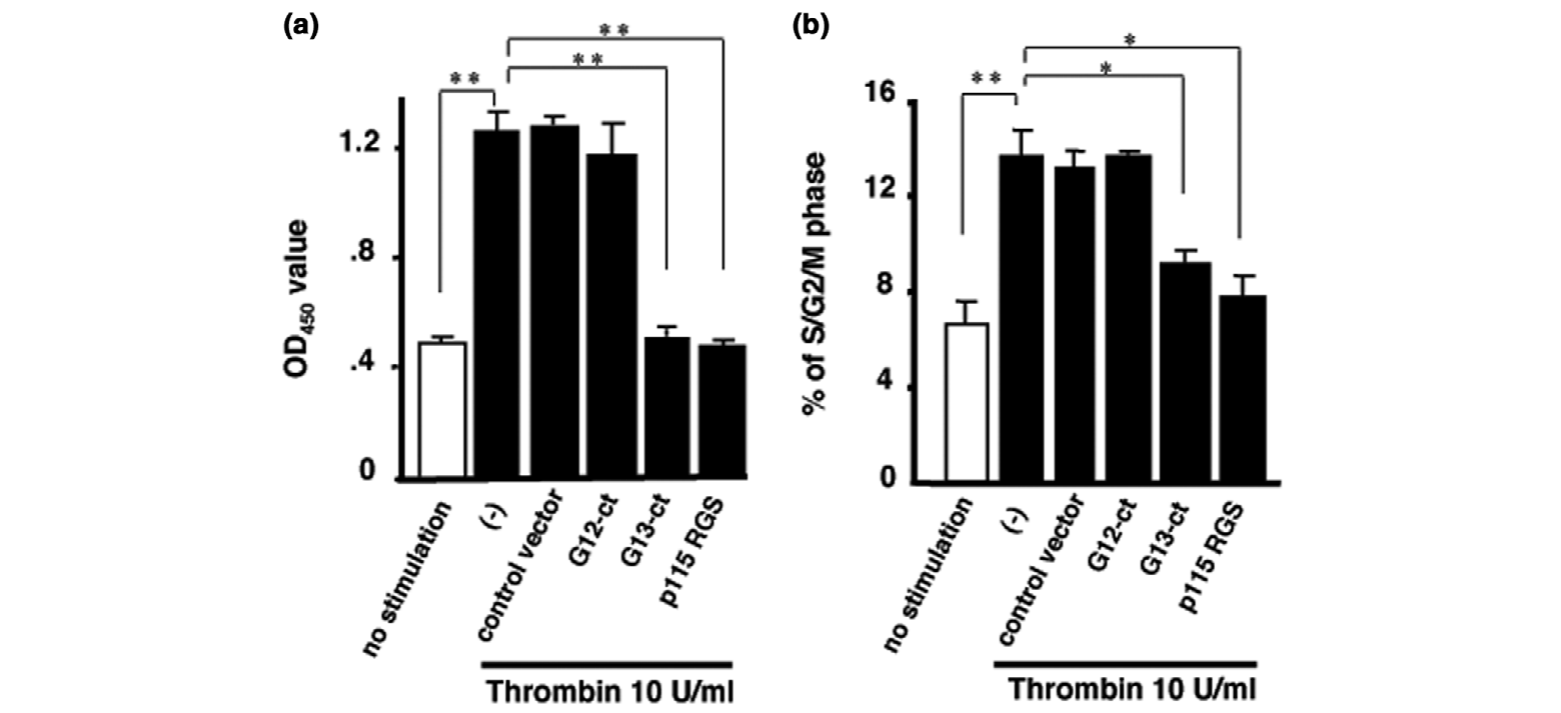
G13 and Rho signaling in thrombin-mediated synovial proliferation and S-phase progression in RA SFs. Rheumatoid arthritis (RA) synovial fibroblasts (SFs) infected or not infected with adenoviruses encoding GFP (control vector), the C-terminal regions of Gα12 (Gα12-ct), Gα13-ct, or p115RGS were cultured for 48 hours in DMEM containing 1% FCS and then stimulated with 10 units/ml thrombin. At 24 hours after the thrombin stimulation, proliferation assay and cell-cycle analysis of RA SFs were performed. **(a) **Effect of Rho signaling inhibition on thrombin-induced cell proliferation. Numbers represent the optical density (OD) as measured by ELISA plate reader at 450 nm. **(b) **Effect of Rho signaling inhibition on thrombin-induced cell-cycle progression. Numbers represent the percentage of cells that exhibited mean channel fluorescence (FL2-H) in the S/G_2_/M phase. Data are expressed as mean ± standard deviation of five experiments, using five independent donors. (-), cells without infection; p115RGS, regulator of G-protein signaling domain of p115Rho guanine nucleotide exchange factor. *P < 0.05, **P < 0.01, in comparison with thrombin stimulation.

Thrombin (10 unit/ml) significantly induced synovial proliferation and S-phase progression in RA SFs infected or not infected with adenoviruses encoding control vector and Gα12-ct (Fig. 5a,b). In contrast, thrombin failed to induce both cell proliferation and S-phase progression in RA SFs expressing Gα13-ct and p115RGS (Fig. 5a,b). These data suggest that Gα13 (but not Gα12) and RhoGEF are involved in signaling via thrombin-stimulation, which leads to synovial proliferation.

### Induction of IL-6 secretion via Rho-mediated signaling in RA SFs

**Figure 6:**
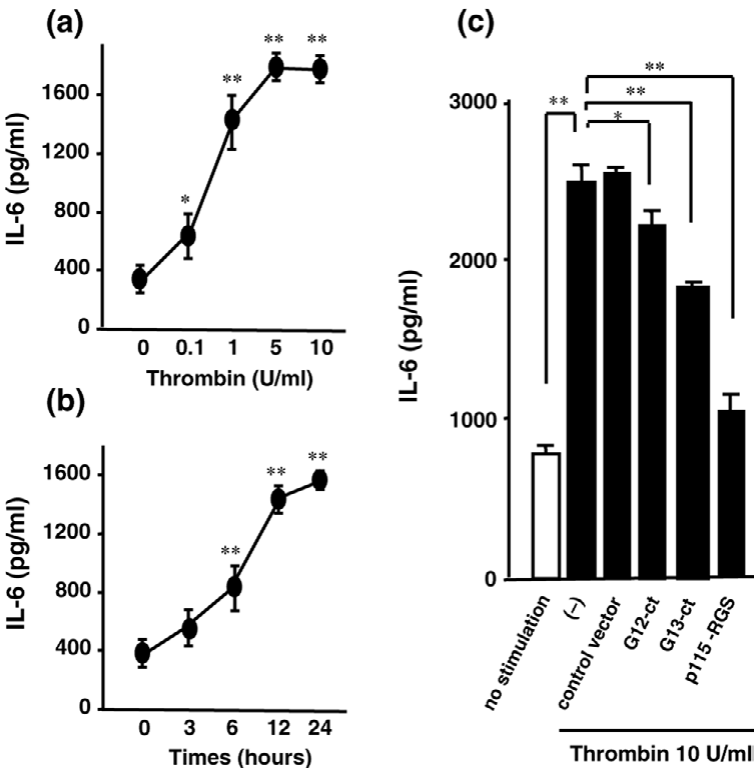
Thrombin induces IL-6 secretion via Rho-mediated signaling in RA synovial fibroblasts. Rheumatoid arthritis (RA) synovial fibroblasts (SFs) were or were not infected with adenoviruses encoding green-fluorescent protein (control vector), the C-terminal regions of Gα12 (Gα12-ct), Gα13-ct, or p115RGS. Cells were cultured for 48 hours in DMEM containing 1% FCS, then stimulated with the indicated amount of thrombin. At 3 to 24 hours after thrombin stimulation, the supernatants of cultured cells were collected and assayed for IL-6 using commercial ELISA kits. **(a) **Dose-dependent IL-6 production by RA SFs at 12 hours after thrombin stimulation. **(b) **Time course of IL-6 secretion by RA SFs stimulated with 10 units/ml thrombin. **(c) **Effect of Rho signaling inhibition on thrombin-induced IL-6 secretion at 12 hours after thrombin stimulation. Data are expressed as mean ± standard deviation of five experiments, using five independent donors. (-), cells without infection; p115RGS, regulator of the G-protein signaling domain of p115Rho guanine nucleotide exchange factor. *P < 0.05, **P < 0.01, in comparison with (a) time 0, (b) no thrombin stimulation, and (c) the indicated data.

Finally, we assessed IL-6 secretion by RA SFs, using ELISA. Fig. 6 shows the concentrations of IL-6 in supernatants of thrombin-stimulated RA SFs. Stimulation with 10 units/ml thrombin significantly increased IL-6 secretion at 6 hours (Fig. 6b), and this effect was dose-dependent (Fig. 6a). The same dose of thrombin produced a significant induction of IL-6 secretion by RA SFs infected or not infected with adenoviruses encoding control vector (Fig. 6c). However, the thrombin-induced IL-6 secretion in RA SFs transfected with Gα12-ct and Gα13-ct was partially reduced and that in RA SFs expressing p115RGS was markedly inhibited (Fig. 6c). These results suggest that thrombin-induced IL-6 secretion by RA SFs is mainly mediated through RhoGEF.

## Discussion

The multiple functions of synovial cells including proliferation, apoptosis, adhesion, and cytokine production are induced by intracellular signaling, which plays a pivotal role in the pathological processes of RA, a representative inflammatory disease. Among the signaling molecules, we document here the relevance of Rho to the pathogenesis of RA synovitis, based on the following results: high expression of thrombin receptor on RA SFs and thrombin markedly increased the proliferation of these cells and progression of the cell cycle to S phase; thrombin induced the activation of Rho; Rho activation as well as proliferation and S-phase progression were completely blocked by either p115RGS or Gα13-ct; and thrombin-induced IL-6 secretion was also reduced by p115RGS and Gα13-ct.

Rho is activated by the G12/13 family of heterotrimeric GTP-binding proteins through the stimulation of GEF activity of p115RhoGEF [12,39]. However, p115RhoGEF can activate as well as inhibit Rho signaling after stimulation of protease-activated receptor-1 [9,41]. p115RhoGEF also contains the regulator of G-protein signaling (RGS) domain at its N terminus, through which it interacts with Gα12/13 and functions as a GTPase-activating protein for G12/13 [39,42]. Furthermore, several groups have reported that C-terminal fragments of Gα12 and Gα13 can inhibit the interaction of receptors with G12 and G13, respectively [33,40,43]. Therefore, we used p115RGS domains and C-terminal fragments of Gα subunits as inhibitors to analyze Gα12- and Gα13-mediated signaling pathways. Using the N-terminal RGS domain of p115RhoGEF, we observed that thrombin activated Rho-dependent signaling and induced synovial proliferation via the G13 pathway.

Previous studies postulated that local fibrin deposition promotes inflammation and tissue destruction, based on the findings of activation of the coagulation system and local generation of fibrin in inflamed arthritic joints [25,44]. Furthermore, recent studies indicate that the coagulation system is closely associated with the inflammation of RA. The level of thrombin, a ligand for G13, is markedly increased in the synovial fluid and tissue of RA patients, compared with those of OA patients, and significantly correlates with RA activity [24]. Our results also indicated that the expression of the thrombin receptor was significantly higher in RA than OA synovial fibroblasts. Since the expression of thrombin receptor is up-regulated by thrombin itself, it is conceivable that up-regulation of thrombin receptor in RA SFs is a natural consequence of exposure to extravasated plasma thrombin and tissue remodeling during the inflammatory response [22,44,45]. Since thrombin induced cell growth and cell-cycle progress of both RA and OA SFs, thrombin-mediated activation of fibroblasts may not be specific for RA SFs. However, responses of RA SFs to thrombin were significantly higher than those of OA SFs, suggesting that the thrombin–Rho pathway could be activated in RA SF.

In the present study, we observed failure of thrombin to induce both cell proliferation and S-phase progression in RA SFs that expressed Gα13-ct and p115RGS, but not Gα12-ct. These data suggest that G13 and p115RhoGEF, which is directly stimulated by G13, are involved in signaling via thrombin-stimulation, subsequent Rho activation, and synovial proliferation.

IL-6 plays an important role in the pathogenesis of RA [46-51], since it is induced by a variety of stimuli such as IL-1 and TNF, is produced abundantly in RA synovium, and is detected at high concentrations in the synovial fluid and serum of RA. Among the various inflammatory cytokines, production of IL-1α, IL-1β, and TNF-α from RA SFs did not change after thrombin stimulation, as reported previously [25]. The obtained results showed that thrombin markedly induced IL-6 secretion from RA SFs. The thrombin-induced IL-6 secretion was completely inhibited in RA SFs expressing p115RGS, whereas it was partially suppressed in the cells that expressed Gα12-ct and Gα13-ct, suggesting the possible existence of another pathway for the activation of RhoGEF during IL-6 secretion in RA SFs.

Considering these findings all together, we conclude that Rho plays a key role in synovial proliferation, S-phase cell-cycle progression, and IL-6 secretion by thrombin-stimulated RA SFs during the pathological process of synovial inflammation. Furthermore, because p115RGS and Gα13 appear to be potent inhibitors of these cellular functions by targeting the thrombin–G13–GEF–Rho pathway, a rational design of future therapeutic strategies for RA synovitis could perhaps include the exploitation of the Rho pathway to directly reduce synovial cell growth *in vivo*.

## Conclusion

Our results indicate that stimulation with thrombin induced proliferation and IL-6 secretion by RA SFs through G13 and Rho pathways and suggest that the G13–GEF–Rho pathway plays an important role in the RA inflammatory process. A rational design of future therapeutic strategies for RA synovitis could perhaps include the exploitation of the Rho pathway to directly reduce synovial cell growth.

## Abbreviations

DMEM = Dulbecco's modified Eagle's medium; ELISA = enzyme-linked immunosorbent assay; FACS = fluorescence-activated cell sorter; FCS = fetal calf serum; Gα12-ct = the carboxy-terminal regions of Gα12; Gα13-ct = the carboxy-terminal regions of Gα13; GAP = GTPase-activating protein; GEF = guanine nucleotide exchange factor; GFP = green-fluorescent protein; GST = glutathione S-transferase; IL = interleukin; mAb = monoclonal antibody; OA = osteoarthritis; p115RGS = regulator of G-protein signaling domain of p115RhoGEF; PBS = phosphate-buffered saline; RA = rheumatoid arthritis; RBD = Rhotekin-Rho-binding domain; RGS = regulator of G-protein signaling; SD = standard deviation; SF = synovial fibroblast; TNF = tumor necrosis factor.

## Competing interests

The author(s) declare that they have no competing interests.

## Authors' contributions

SN designed the experimental design of the study, carried out the experiments, and drafted the manuscript. HK provided adenovirus and participated in the preparation of the manuscript. KS participated in the experimental design of the study. AM performed statistical analyses and participated in the design of the study. YT conceived of the study, participated in its design and coordination and helped to draft the manuscript. All authors read and approved the final manuscript.
